# Enriched environment and masticatory activity rehabilitation recover spatial memory decline in aged mice

**DOI:** 10.1186/1471-2202-14-63

**Published:** 2013-06-28

**Authors:** Fabíola de Carvalho Chaves de Siqueira Mendes, Marina Negrão Frota de Almeida, André Pinheiro Gurgel Felício, Ana Carla Fadel, Diego de Jesus Silva, Thaíssa Gomes Borralho, Rodrigo Perez da Silva, João Bento-Torres, Pedro Fernando da Costa Vasconcelos, Victor Hugh Perry, Edson Marcos Leal Soares Ramos, Cristovam Wanderley Picanço-Diniz, Marcia Consentino Kronka Sosthenes

**Affiliations:** 1 Universidade Federal do Pará/UFPA, Instituto de Ciências Biológicas, Laboratório de Investigações em Neurodegeneração e Infecção, Hospital Universitário João de Barros Barreto, Rua dos Mundurucus, 4487 – Guamá Belém, Pará, Brasil; 2 Instituto Evandro Chagas/ IEC, Departamento de Arbovirologia e Febres Hemorrágicas, Ananindeua, Pará, Brasil; 3 Southampton Neuroscience Group, School of Biological Sciences, University of Southampton, Southhampton, UK; 4 Universidade Federal do Pará/UFPA, Instituto de Ciências Exatas e Naturais, Laboratório de Sistema de Informação e Georrefrenciamento, Pará, Brasil

**Keywords:** Mastication, Enriched environment, Masticatory rehabilitation, Spatial learning, Morris water maze

## Abstract

**Background:**

To measure the impact of masticatory reduction on learning and memory, previous studies have produced experimental masticatory reduction by modified diet or molar removal. Here we induced spatial learning impairment in mice by reducing masticatory activity and then tested the effect of a combination of environmental enrichment and masticatory rehabilitation in recovering spatial learning at adulthood and in later life. For 6 months (6M) or 18 months (18M), we fed three groups of mice from postnatal day 21 respectively with a hard diet (HD) of pellets; pellets followed by a powdered, soft diet (HD/SD, divided into equal periods); or pellets followed by powder, followed by pellets again (HD/SD/HD, divided into equal periods). To mimic sedentary or active lifestyles, half of the animals from each group were raised from weaning in standard cages (impoverished environment; IE) and the other half in enriched cages (enriched environment; EE). To evaluate spatial learning, we used the Morris water maze.

**Results:**

IE6M-HD/SD mice showed lower learning rates compared with control (IE6M-HD) or masticatory rehabilitated (IE6MHD/SD/HD) animals. Similarly, EE-HD/SD mice independent of age showed lower performance than controls (EE-HD) or rehabilitated mice (EE-HD/SD/HD). However, combined rehabilitation and EE in aged mice improved learning rate up to control levels. Learning rates did not correlate with swim speed.

**Conclusions:**

Reduction in masticatory activity imposed on mice previously fed a hard diet (HD/SD) impaired spatial learning in the Morris water maze. In adults, masticatory rehabilitation recovered spatial abilities in both sedentary and active mice, and rehabilitation of masticatory activity combined with EE recovered these losses in aged mice.

## Background

The proportion of the elderly population that experiences dementia is rapidly increasing, and both human and animal studies have indicated a relationship between reduced masticatory function (e.g., from occlusal disharmony) in elderly individuals and cognitive impairment [1,2]. In addition, data from human and animal reports reveal that an impoverished environment (IE) is associated with aggravation of aging-related cognitive decline; for a recent review see [3]. Indeed, compared with geriatric persons with poor levels of physical and social activities, exercise programs for institutionalized older people improve cognitive function [4-6]. Consistent experimental data from aged mice and rats maintained in IEs also point to spatial memory impairments in Morris water maze tests [7,8]. Water maze tests require acquisition and retrieval of spatial information [9], and this hippocampal-dependent task [10,11] can be impaired by a variety of structural/functional changes including aging [12], IE [3], and occlusal disharmony [2,13].

Human epidemiological studies [14-16] and experimental data from rodents [17-19] also show direct correlations between aging, masticatory imbalances (e.g., occlusal disharmony), and cognitive decline, but with no references to environmental conditions. Experimental approaches imposing masticatory deprivation, like tooth-loss [20], long-term soft diet [21], or bite-rise occlusion, suggest a behavioural displacement, but more research is needed into the effects of rehabilitation. Here we examined outcomes at the intersection of masticatory deprivation, environmental conditions, and spatial memory impairments in adult (6-month-old; 6M) and aged (18-month-old; 18M) mice. To assess the effects of masticatory rehabilitation on the Morris water maze task, we used different sequences of hard diet (HD) and soft diet (SD) on the aged and young mice and included an environmental component with an IE or enriched environment (EE). To mimic masticatory rehabilitation, we fed some animals with HD followed by SD, with a return to HD, and compared them to animals without rehabilitation (HD/SD).

## Results

Masticatory reduction, environmental changes, and water maze tests at 6 and 18 months.

### Learning rate

**Figure 1:**
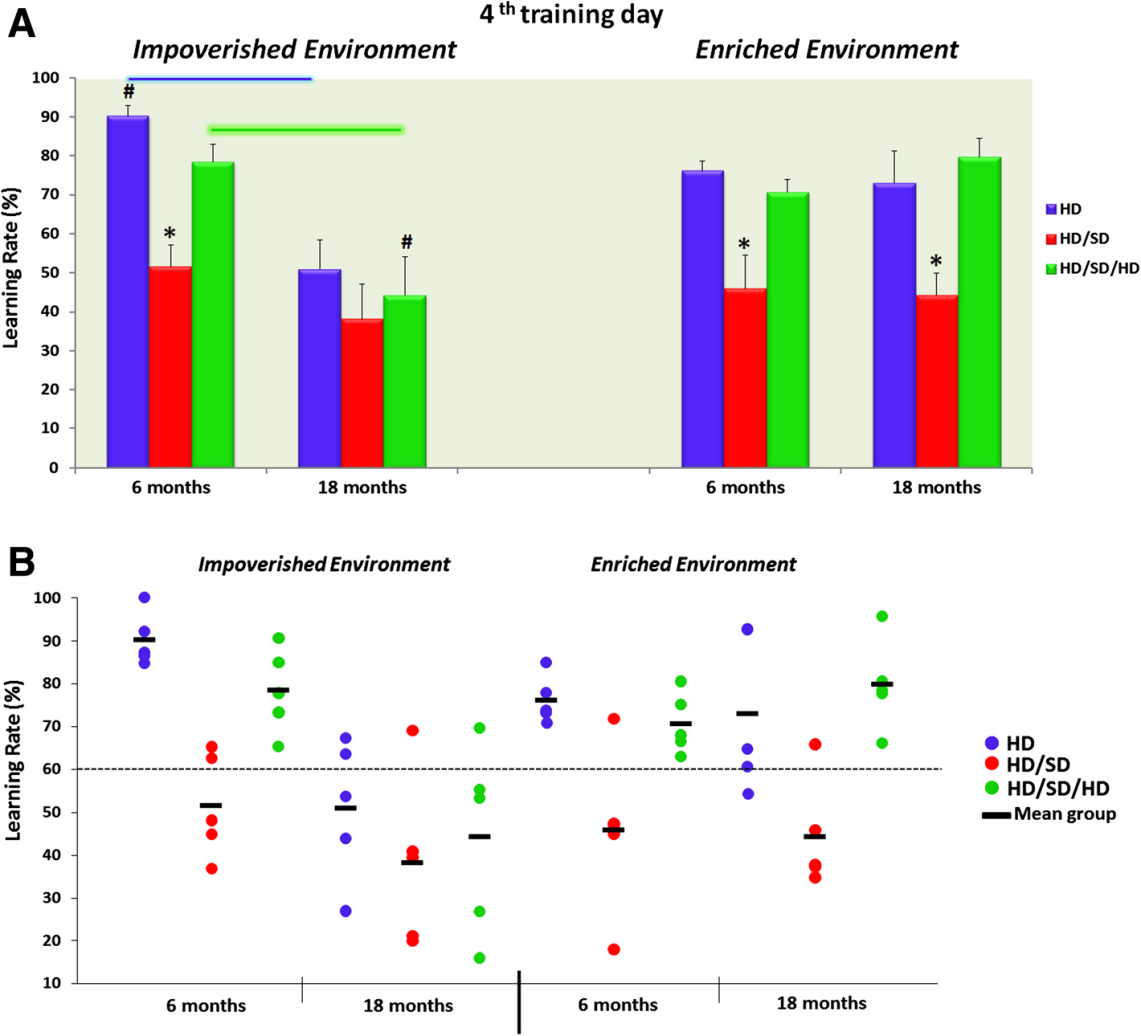
**Learning rate on water maze test under different diet regimes, ages, and environments.** Graphic representation of diet regime, age, and environmental influences on learning rate of the best five performances of each experimental group on the fourth training day. In the top panel (**A**), indicated in different colours, are the mean values and standard errors of the learning rates with respective significance values, and in the bottom panel (**B**) are the individual performances (coloured, solid circles) and mean groups (dark dashes between solid circles). (*) indicates significant differences in learning rates between different diet regimes, (#) significant differences in learning rates between environments, and coloured line connectors differences in learning rates between age.

Figure 1 depicts the diet regime, age, and environmental influences on learning rate of the best five performances of each experimental group on the fourth training day. The top panel (Figure 1A) indicates the significant between-group differences in the learning rates, and the bottom (Figure 1B) panel shows individual performances and group means. Note that at 6M, impoverished conditions associated with a reduction in masticatory activity (HD/SD) were also associated with lower learning rate values compared with control (HD) or masticatory rehabilitated (HD/SD/HD) mice. On the other hand, spatial learning performance was impaired in aged IE mice regardless of diet regime but less so in the rehabilitated group. Similarly, EE-HD/SD animals independent of age showed lower performance than EE controls (HD) or rehabilitated mice (EE-HD/SD/HD). However, rehabilitation + EE in aged mice improved learning rates up to EE control levels. Indeed, on the fourth training day, learning rate was influenced by diet regime (F_(2,48)_ = 21.2, p < 0.000001) and age (F_(1,48)_ = 13.6, p < 0.000567) with interactions between environment and age (F_(1,48)_ = 16.7, p < 0.00164). Pairwise comparisons (Tukey Honestly Significant Difference) showed significant differences between mean values of IE6M-HD (90.19 ± 2.74, mean ± S.E.) and IE6M-HD/SD (51.60 ± 5.40) (t(8) = 6.38, p < 0.0002); IE6M-HD and IE6M-HD/SD/HD (78.49 ± 4.43) (t(8) = 2.25, p < 0.05); IE6M-HD and IE18M-HD (51.02 ± 7.26) (t(8) = 5.05, p < 0.001); IE6M-HD and EE6M-HD (76.14 ± 2.48) (t(8) = 3.80, p < 0.005); IE6M-HD/SD and IE6M-HD/SD/HD (t(8) = 3.85, p < 0.005); IE6M-HD/SD/HD and IE18M-HD/SD/HD (44.25 ± 9.89) (t(8) = 3.16, p < 0.013); and IE18M-HD/SD/HD and EE18M-HD/SD/HD (79.75 ± 4.77) (t(8) = 3.23, p < 0.01). For EE-only animals, we identified the following values: EE6M-HD and EE6M-HD/SD (45.91 ± 8.53) (t(8) = 3.40, p < 0.009); EE6M-HD/SD and EE6M-HD/SD/HD (70.66 ± 3.19) (t(8) = 2.72, p < 0.03); EE18M-HD (73.03 ± 8.22) and EE18M-HD/SD (44.35 ± 5.70) (t(8) = 2.87, p < 0.02); and EE18M-HD/SD and EE18M-HD/SD/HD (t(8) = 4.76, p < 0.001).

### Swim speed and distance travelled

**Figure 2:**
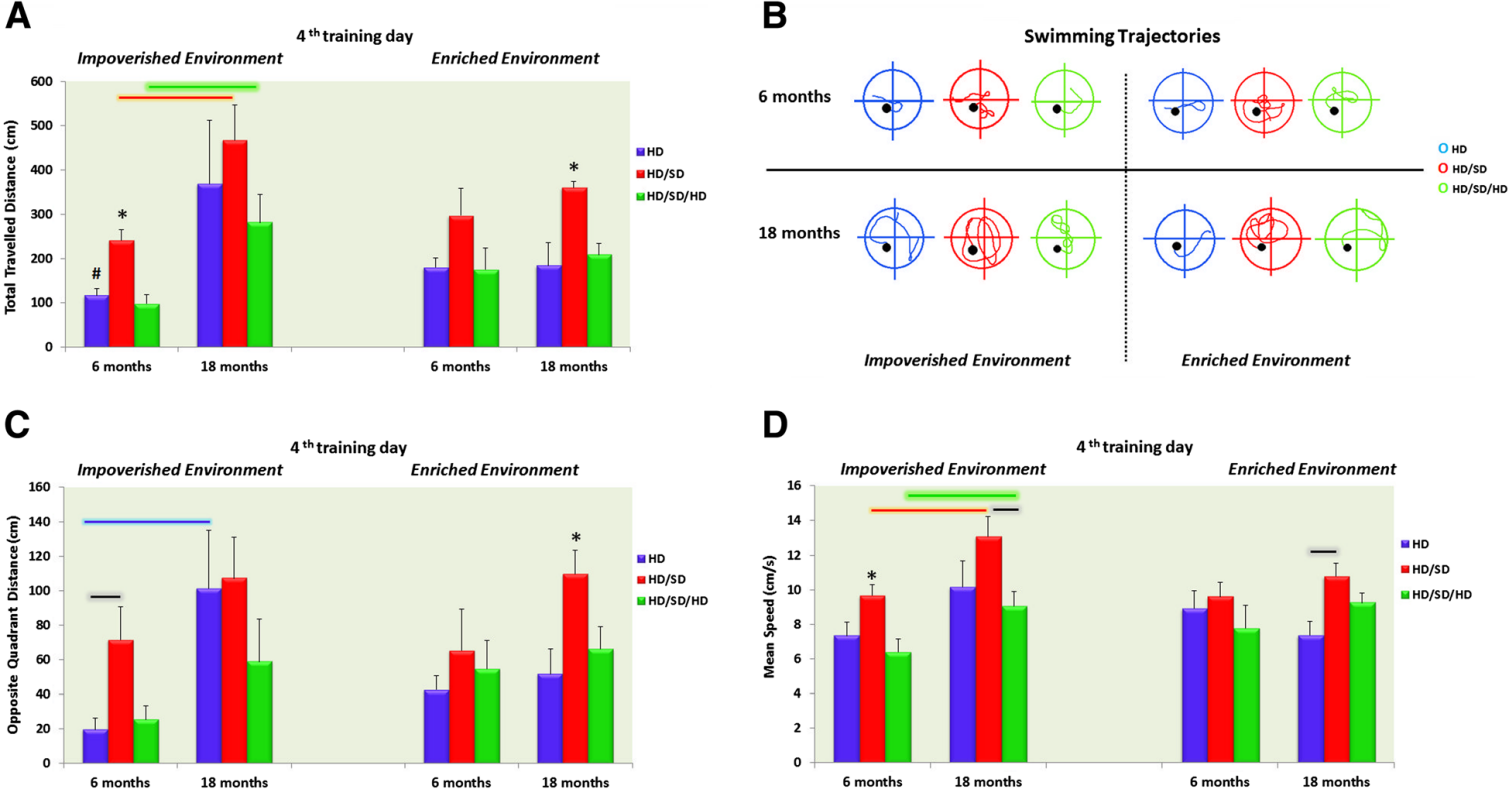
**Total and opposite quadrant travelled distances, representative swimming trajectories, and swimming speed.** In the top panels, indicated in different colours, are the mean values and standard errors of the total distances (**A**) and representative swimming trajectories of each group of animals closer to the mean distance group (**B**). In the bottom panels are the mean values of opposite quadrant distances (**C**) and swimming speeds (**D**). All data plots are expressed as a function of age, environment, and diet regimes. As expected, HD/SD groups, independent of age or environment, swam longer distances both in total and in the opposite quadrant than all other groups. (*) indicates significant differences between different diet regimes, (#) significant differences between environments, and coloured line connectors differences between ages, and the edges of the black line link differences between diet.

Because a reduction in escape latency during training might simply suggest that mice learned to swim faster across days and or abandoned the thigmotactic strategy that is preferred during the very first days of training, in Figure 2 we show total distance travelled (path length) (Figure 2A) and respective mean trajectories (Figure 2B); distance travelled on the quadrant opposite the platform (Figure 2C); and the average swim speed (Figure 2D). Three-way ANOVA of these data revealed that distance travelled was influenced by age (F_(1,48)_ = 13.6, p < 0.0006) and diet (F_(2,48)_ = 7.55, p < 0.001) with significant interaction between environment and age (F_(1,48)_ = 7.40, p < 0.009). As expected, the best performances in learning rates were associated with shorter distances travelled. Indeed, pairwise comparisons showed that IE6M-HD/SD (241.36 ± 23.77 cm, mean ± S.E.) travelled longer distances than IE6M-HD (116.23 ± 16.07) (t(8) = 4.36, p < 0.002) and IE6M-HD/SD/HD (98.03 ± 19.55) (t(8) = 4.66, p < 0.002), but shorter than IE18M-HD/SD (467.93 ± 79.75) (t(8) = 2.72, p < 0.026) and IE18M-HD/SD/HD (281.14 ± 63.03) (t(8) = 2.78, p < 0.02). Consistent with learning rate results, IE6M-HD also travelled shorter distances than EE6M-HD (179.87 ± 21.43) (t(8) = 2.38, p < 0.045). In addition, EE18M-HD/SD (360.23 ±13.21) swam longer distances to find the platform than EE18M-HD (183.82 ± 52.02) (t(8) = 3.29, p < 0.01) or EE18M-HD/SD/HD (207.83 ± 26.32) (t(8) = 5.18, p < 0.0008).

In keeping with these findings, the distance travelled in the opposite quadrant to the platform was also influenced by age (F_(1,48)_ = 10.9, p < 0.0019) and diet (F_(2,48)_ = 4.83, p < 0.01), but no interactions were identified. Pairwise analysis demonstrated that IE6M-HD (19.69 ± 6.54, mean ± S.E.) swam less in the opposite quadrant than IE6M-HD/SD (71.67 ± 19.30) (t(8) = 2.55, p < 0.03) and IE18M-HD (101.45 ± 34.03) (t(8) = 2.36, p < 0.046). In addition, EE18M-HD/SD (109.78 ± 14.11) swam longer distances in the opposite quadrant than EE18M-HD (51.69 ± 14.62) (t(8) = 2.86, p < 0.02) and EE18M-HD/SD/HD (66.19 ± 13.03) (t(8) = 2.27, p < 0.05).

Finally, three-way ANOVA indicated that swim speed was affected by age (F_(1,48)_ = 9.08, p < 0.004) and diet (F_(2,48)_ = 9.26, p < 0.0004) with significant interaction between environment and age (F_(1,48)_ = 5.51, p < 0.023). However, pairwise comparisons showed that IE6M-HD/SD (9.67 ± 0.63 cm/s, mean ± S.E.) swam faster than IE6M-HD (7.35 ± 0.78) (t(8) = 2.31, p < 0.05) and IE6M-HD/SD/HD (6.39 ± 0.75) (t(8) = 3.33, p < 0.01) but slower than IE18M-HD/SD (13.09 ± 1.13) (t(8) = 2.63, p < 0.03). In addition, IE6M-HD/SD/HD swam slower than IE18M-HD/SD/HD (9.05 ± 0.83) (t(8) = 2.39, p < 0.04), and IE18M-HD/SD swam faster than IE18M-HD/SD/HD (t(8) = 2.87, p < 0.021). However, swim speed did not change in animals raised in enriched conditions with a single exception: EE18M-HD (7.36 ± 0.79) swam slower than EE18M-HD/SD (10.77 ± 0.78) (t(8) = 3.07, p < 0.02).

Taking these results together, learning rates seem to be directly related to distance travelled, but swim speed values do not, suggesting that the behavioural effects are not the result of sensorimotor changes and that masticatory activity seems to affect spatial learning in the Morris water maze test. The findings also indicate that a combination of increased masticatory activity and EE in animals with a previous reduction in masticatory activity may benefit both young and aged mice.

### Body weight and diet regimes

To detect a possible influence of diet regimes and environment on body weight, we weighed all animals after behavioural tests. One-way ANOVA revealed significant influence of diet regimes on body weight of IE animals (F_(2, 12)_ = 16.04, p < 0.0006), and IE6M-HD/SD (52.84 ± 2.45 g, mean ± S.E.) weighed significantly less than IE6M-HD (75.96 ± 3.69) or IE6M-HD/SD/HD (71.56 ± 2.93) mice (Bonferroni post-tests, p < 0.05). No body weight differences were detected in EE animals. We did find a single significant inverse correlation between body weights and performance in the water maze in IE6M-HD/SD mice (Pearson’s coefficient = −0.99; R^2^ = 0.99, p = 0.0007).

In addition, t-tests revealed no significant differences between body weights of IE6M-HD/SD and EE6M-HD/SD mice (two-tailed t-test, p = 0.38) and no differences in learning rates. Based on this finding, we speculated that the sedentary IE lifestyle is associated with being overweight and that the HD/SD diet regime in IE may reduce food intake. Observing the active lifestyle of EE animals (EE6M-HD vs IE6M-HD, two-tailed t-test, p =0.0002; EE6M-HD/SD/HD vs IE6M-HD/SD/HD, two-tailed t-test, p = 0.0049), we noted that it reduced weight gain independent of the diet regime. However, these differences disappeared after 18M in all diet regimes.

## Discussion

In the present report, we tested the hypothesis that a combination of masticatory rehabilitation and EE would recover impaired spatial learning induced by a combination of IE and reduced masticatory activity. To mimic sedentary or active lifestyles, respectively, we raised mice from weaning onwards in either IE or EE. To mimic a reduction in masticatory activity, we fed a soft diet to mice previously fed with a hard diet (HD/SD) and compared water maze performances of this group with two other groups fed with continuous hard diet (HD – control group) or with a sequence of hard, soft, and hard diets (HD/SD/HD – rehabilitated group). We found that a reduction in masticatory activity and IE impaired spatial learning on the Morris water maze and a combination of masticatory rehabilitation and EE recovered spatial learning abilities of both adult and aged mice.

### Aging cognitive decline and environment

We tested adult and aged mice raised in IE or EE for spatial memory and learning using the water maze paradigm. The results revealed that all IE and EE adult mice could learn and remember the position of the hidden platform after four training days; however, only aged EE mice met the criterion of 60% or more in learning rate (EE18M-HD = 73.03 ± 8.22). Indeed, the aged IE mice learning rate was on average near the chance level (IE18M-HD = 51.02 ± 7.26). These findings suggest that after 6 months of impoverished conditions, the hippocampal requirements for spatial learning were spared in young mice and could be activated after training. As expected, memory capabilities became worse when advanced age was combined with IE; under these conditions, age-related spatial learning decline was aggravated. In contrast, EE-raised aged mice exhibited relatively unimpaired spatial learning in water maze tests, suggesting that the consolidation and retrieval mechanisms for these memories were spared under enriched conditions. These observations replicate previous results of ours and others in mice and rats undergoing spatial learning and memory tests [7,22-29].

More recently, we investigated the impact of institutionalization on aged humans, living sedentarily under poor conditions of cognitive and motor–sensory stimulation (impoverished-like conditions). Participants underwent neuropsychological tests, and outcomes were compared to those of an aged control group living in the community with their families (enriched-like conditions). Our findings revealed significantly higher scores among community-living participants. Consistent with this finding, institutionalized patients after 6 months of motor–sensory and cognitive stimulation (enriched-like environment) experienced significant improvement in their performances on neuropsychological tests [30].

### Rehabilitation of masticatory function to prevent adult and aging-related spatial learning losses

A number of studies using a variety of behavioural assays, including the Morris water maze [18,19,31-36], passive avoidance [37], and radial arm maze [20,38,39], indicate that long-term soft-diet feeding or extraction of molar teeth results in learning and memory deficits [40]. Recent studies with the Morris water maze demonstrated that mice fed a hard diet required significantly less time to reach the platform than experimental mice and spent significantly more time in the former platform area, suggesting that hard-diet feeding is associated with improved spatial memory [41]. In addition, one-year-old [35] or 18-month-old [36] adult mice fed with a soft diet, compared to a solid-diet group, showed lower spatial performances on the Morris water maze. Similarly, SAMR1 and SAMP8 mice fed a solid diet performed better in the eight-arm radial maze than mice fed a powdered diet [39]. SAMP8 mice reach adult maturity at 6 months of age when learning and memory deficits become apparent in a variety of behavioural tests [42-45] and when reduced masticatory activity accelerates age-related learning and memory decline [40]. Occlusal disharmony also aggravates age-dependent deficits in spatial learning in the Morris water maze [17,46-49]; see [13,40] for recent reviews. Moreover, monkey studies demonstrate that when occlusal disharmony is removed, the associated stressful response disappears [50].

Here the results add a new piece of information, demonstrating a vigorous effect of diet changes on spatial learning in the Morris water maze after a reduction in masticatory activity imposed with a soft diet on mice previously fed a hard diet. We also demonstrated that these spatial learning losses can be promptly recovered by masticatory rehabilitation at adulthood (6M) but that aged mice (18M) require a combination of environmental stimuli and masticatory rehabilitation to obtain similar effects. Because the Morris water maze is a hippocampal-dependent task, we suggest that rehabilitation of masticatory activity in adult mice and a combination of EE and masticatory rehabilitation in aged mice may rescue hippocampal function.

We emphasize that the animals were deprived of their masticatory activity but not fully suppressed in an effort to simulate the human condition. Taking all of the results together, we can speculate that oral rehabilitation and sensory–motor and cognitive stimulation may help protect human subjects from age-related cognitive decline and that these interventions would be more effective if implemented as early as possible.

## Conclusions

An imposed reduction in masticatory activity by administration of a soft diet to sedentary mice previously fed a hard diet impaired spatial learning in the Morris water maze. The rehabilitation of masticatory activity of these deprived mice at adulthood, independent of environment, recovered spatial learning losses, and a combination of enriched environment and rehabilitation benefited significantly both young and aged mice. We are now investigating the cellular and molecular changes associated with spatial learning recovery after masticatory rehabilitation.

## Methods

All animals, female albino Swiss mice, were maintained in accordance with the guidelines published by the National Institutes of Health (*Guide for the Care and Use of Laboratory Animals*). The experimental protocol was tested and approved prior to study initiation by the Ethics Committee on Experimental Animal Research (from the Institute of Biological Sciences, Federal University of Pará, Brazil, CEPAE-UFPA:BIO004-09).

### Environment, age, diet regimes, and experimental groups

**Figure 3:**
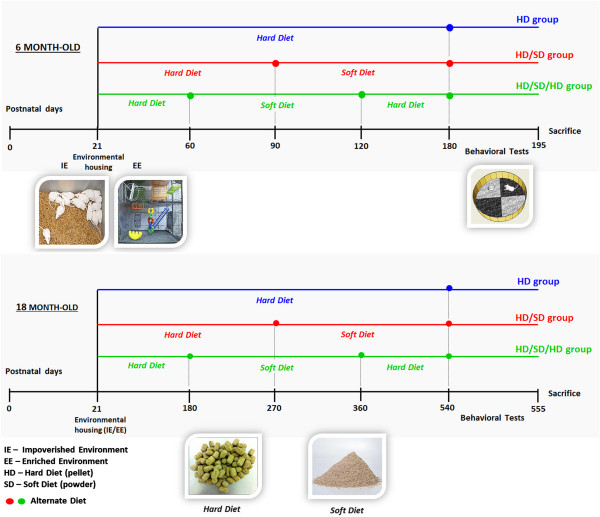
**Experimental timeline.** Female albino Swiss mice were housed either in impoverished or enriched environments (middle panel left) from postnatal days 21 until 180 (6 months old; 6M) or 540 (18 months old; 18M), under one of the following diet regimes: HD groups correspond to continuous pellet diets; HD/SD regimes for 6 or 18 months correspond to alternating pellet and powder diet every 3 or 9 months, respectively; HD/SD/HD regimes for 6 or 18 months correspond to sequences of pellet, powder, and pellet food every 2 or 6 months, respectively. All 6M and 18M animals were submitted to the Morris water maze (middle panel, right) between 180 and 195 and between 540 and 555 postnatal days, respectively. Bottom panel: pellet (hard diet) and powder (soft diet) food. Time is represented in days after birth and increases from left to right.

The EE consisted of two-level wire cages (100×50×100 cm) equipped with ropes, rod bridges, tunnels, running wheels, and toys. Toys were made of different forms of plastic, wood, and metal of different colours and were changed periodically. Each EE cage housed 20 young and aged mice. Water and food were delivered to the top and bottom levels, respectively. This arrangement obliged mice to move from one compartment to another to drink and eat. Impoverished conditions comprised plastic cages (32×45×16.5 cm) without equipment or toys and covered by metal grids. The IE mice were maintained in groups of six until sacrifice. Each IE cage housed 9 young or aged mice. Animals had free access to food and water and were raised at a controlled room temperature (23 ± 1°C) and 12-hour light–dark cycle. Figure 3 depicts the experimental timeline.

All animals from IE or EE cages were organized into six groups according to diet regime (HD, HD/SD, or HD/SD/HD) and age (6 or 18 months). Animals were housed either in enriched or impoverished environments from postnatal day 21 onwards, under one of the following diet regimes: IE6M (HD, n = 28; HD/SD, n = 15; HD/SD/HD, n = 18), EE6M (HD, n = 27; HD/SD, n = 11; HD/SD/HD, n = 11), IE18M (HD, n = 19; HD/SD, n = 11; HD/SD/HD, n = 12), and EE18M (HD, n = 18; HD/SD, n = 25; HD/SD/HD, n = 27). HD groups were fed a continuous pellet diet; HD/SD regimes for 6 or 18 months followed an alternating pellet and powder diet every 3 or 9 months, respectively; and HD/SD/HD regimes for 6 or 18 months followed sequences of pellet, powder, and pellet food every 2 or 6 months, respectively (Figure 3). All animals were albino Swiss female mice.

### Behavioural tests

At corresponding time points (6 or 18 months), all groups were trained in the water maze paradigm [9], adapted for mouse dimensions. The circular pool and platform were 101 and 13 cm in diameter, respectively; the platform was 1 cm below the water surface. To occlude the platform, the pool was filled with black water (22 ± 2°C) coloured with a non-toxic dye. The first day of water maze training was dedicated to adapting the animal to the aquatic labyrinth. In the remaining 5 days, animals were tested once per day in three trials. Three permanent external cues were used during all day tests, and luminance level in the pool was kept constant between 4–5 cd/m^2^. The enter points in the pool, based on dividing this apparatus according to cardinal points and thus into four equal quadrants, were semi-randomized, and we systematically avoided repeating entry at the same point more than twice. In each trial, the animals were allowed three trials of 60 s each to find the hidden platform; trials were separated by intervals of 30 s, and the task was considered complete when they found and remained on the platform for 10 s.

Any-Maze® tracking software was systematically used to precisely record the position of the mouse throughout the test. From this detailed positional information, we estimated for each animal a daily average value of escape latencies, travelled distances (path length), average swim speed, and time spent in the quadrant opposite to the hidden platform. The learning rate for the water maze was assessed by the ratio between escape latencies to find the platform on the first and subsequent test days (days 2–5) using the following equation: C = (L_1_-L_N_)/ (L_1_+L_N_), where C is a contrast index to express the learning rate and L_1_ and L_N_ are the escape latencies to find the platform on the first and the subsequent test days, respectively. This equation was systematically applied every training day using the escape latency mean value of the daily three trials of each animal. Thus, four values of contrasts were obtained for each animal (L_1_/L_2_, L_1_/L_3_, L_1_/L_4_, and L_1_/L_5_), and a learning rate curve for five training days could be plotted for each animal. To express the learning rate in percentage values for each day, we selected from all experimental groups, independent of the diet regimen, age, or environment, the higher contrast value for each day and estimated the remaining contrast values as follows: C (%) = [(L_1_-L_N_)/(L_1_+L_N_)] * 100 / C_max_, where C_max_ corresponds to the best performance on that day in comparison with the first day and L_1_ and L_N_ correspond to the escape latency in the first and subsequent days, respectively. Because the fourth training day showed the highest number of animals with a learning rate equal to or above 60% in all experimental groups, we selected this training day for comparative analysis between groups. To this end, we chose the five best performances of each experimental group from that day, considering that these five best specimens showed consistent learning (C (%) > 0, at the fourth training day and on at least two more test days, or C (%) > 0, at training days 4 and 5) and applied three-way ANOVA, followed by Tukey post-tests, with differences between groups accepted as significant at a 95% confidence level (p < 0.05). The contrast index has been previously applied to water maze results [51,52] to normalize the learning curve to each individual’s performance, thus accounting for the variation in performances among individuals.

## Competing interests

The authors declare that they have no competing interests.

## Authors’ contributions

FCCSM, MNFA, APGF, ACF, DJS, TGB, RPS, CWPD, and MCKS conceived the study, participated in the experimental design, and drafted the manuscript. FCCSM, MNFA, APGF, ACF, DJS, TGB, RPS, CWPD, and MCKS performed the experiments and analysed and interpreted the data. FCCSM, JBT, PFCV, VHP, EMLSR, CWPD, and MCKS participated in the data analysis, were involved in drafting the manuscript, and made important intellectual contributions. All authors read and approved the final manuscript.
